# Supplementary material for: Regulatory variation controlling architectural pleiotropy in maize
Source: Nat Commun. 2025 Mar 3;16:2140. doi: 10.1038/s41467-025-56884-w (PMC11876617; doi:10.1038/s41467-025-56884-w)
Supplement: Supplementary file 1 — Supplementary Information [file 41467_2025_56884_MOESM1_ESM.pdf]

Regulatory variation controlling architectural pleiotropy in maize

Bertolini *et al.*

Supplementary Note 1. Biology behind the mutants used in this study.

The *liguleless1 (lg1)* gene encodes a SQUAMOSA BINDING PROTEIN (SBP) TF and loss-of function mutants completely abolish ligule and auricle formation, yet maintain a clear blade sheath boundary [1,2]. In *liguleless2* (*lg2*) mutants, the blade-sheath boundary is more diffuse and reduced auricle tissue is retained at the leaf margins. This suggests that LG2, a bZIP TF, may be involved in defining the blade-sheath boundary [3] and that *lg1* is required for proper ligule differentiation [4].

During early tassel development, the indeterminate inflorescence meristem initiates a defined number of indeterminate axillary branch meristems at its flanks, which produce the long tassel branches, and then abruptly shifts to initiating short branches [5]. The *ramosa1* (*ra1*) gene, encoding a C2H2 TF specific to panicoid grasses, controls this process by imposing determinate fate on axillary meristems in a spatiotemporal manner [6]. The RA1 protein accumulates adaxial to the base of axillary meristems that will produce short branches [6]. Prior to RA1 expression, the LG1 protein accumulates in an overlapping domain at the base of the long branch meristems, but not the short ones, and RA1 can bind and negatively modulate *lg1* expression based on ChIP-seq and transcriptome studies [7].

Since RA1 is an inflorescence-specific TF, its regulation of *lg1* is not present in developing ligules. Similarly, WAVY AURICLE BLADE1 (WAB1), a TCP family TF, is a tissue specific factor that promotes expression of *lg1* in the tassel. In *Wab1* gain-of-function mutants, LG1 over-accumulates at the base of tassel branches resulting in wider branch angles and is ectopically expressed in the leaf, causing aberrant ligule tissue in the sheath [8]. Loss-of-function *wab1* mutants (allelic to *branch angle defective1* (*bad1*)), have more upright tassel branches [8,9]. Another branching mutant, *ramosa2* (*ra2*), which encodes a LATERAL ORGAN BOUNDARY (LOB) domain TF [10], acts upstream of *wab1*/*bad1* and in a different pathway than *lg1* [9]. Tassel branches of *ra2* mutants are very upright.

RNAi knockdown lines of the BR receptor kinase, BRASSINOSTEROID INSENSITIVE 1 (BRI1), an initiator of BR signaling, and BR INSENSITIVE 2 (BIN2), a GSK3-like kinase and negative regulator of BR transcriptional response, show opposite architectural phenotypes. The *bri1-RNAi* mutant significantly knocked down *zmbri1* expression as well as expression of four other paralogs, causing decreased BR signaling; ligule and auricles are abnormal and leaves were more upright, and more upright tassel branches [11]. *bin2-RNAi* plants have wide open leaves due to expanded auricles and long, open tassel branches [12].

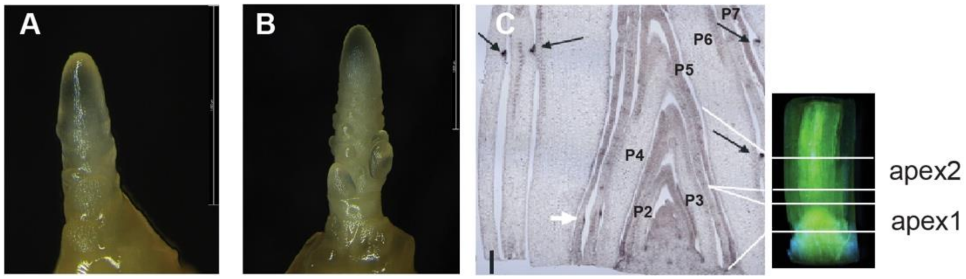

Supplementary Fig. 1. Overview of biological samples collected for this study.
Representative images of hand-dissected tassel primordia from B73 control plants at **A**. stage 1, which captures branch meristem initiation and **B**. stage 2, which captures branch outgrowth. Scale bars =1,000 µm. C. Longitudinal section through the shoot apical meristem (SAM) of a B73 control plant depicting the microscopic view of the shoot apex to the right. *In situ* hybridization with a *liguleless1* (*lg1*) anti-sense probe marks developing ligules. Two sections from the shoot apex were sampled: shoot apex 1 includes the SAM and cells pre-patterned to be ligule and shoot apex 2 excludes the SAM and enriches for developed ligule. Leaf primordia are designated according to plastochron number (P). White arrow denotes the pre-ligular band while black arrows indicate the developing ligule on P7 and successive leaf primordia.

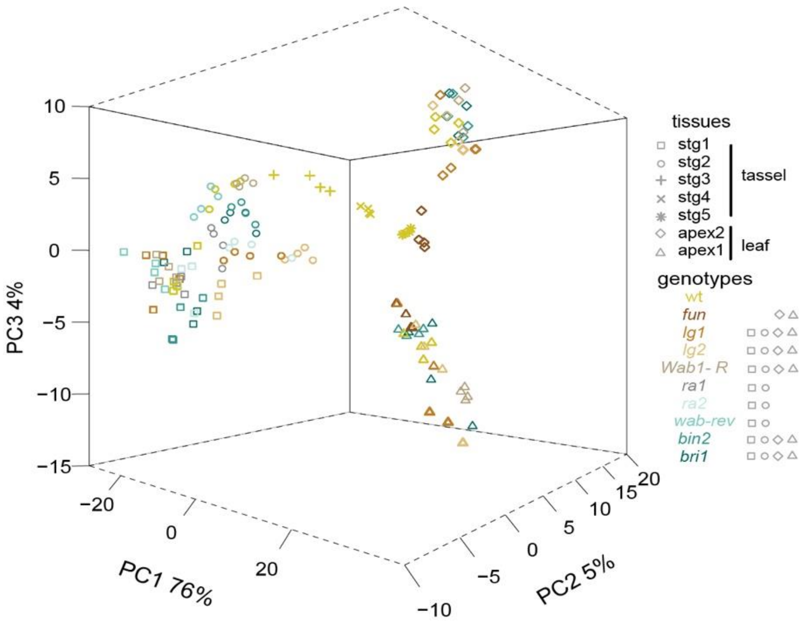

Supplementary Fig. 2. Principal component analysis of the expression dataset.
The image shows the three principal components in 3-dimensional space of the entire transcriptional dataset (*n*=140) generated from the two tassel primordia developmental stages and shoot apex sections. Genotypes are marked by different colors and tissue type by different symbols. The principal component analysis was calculated based on the top 500 dynamically expressed protein-coding genes.

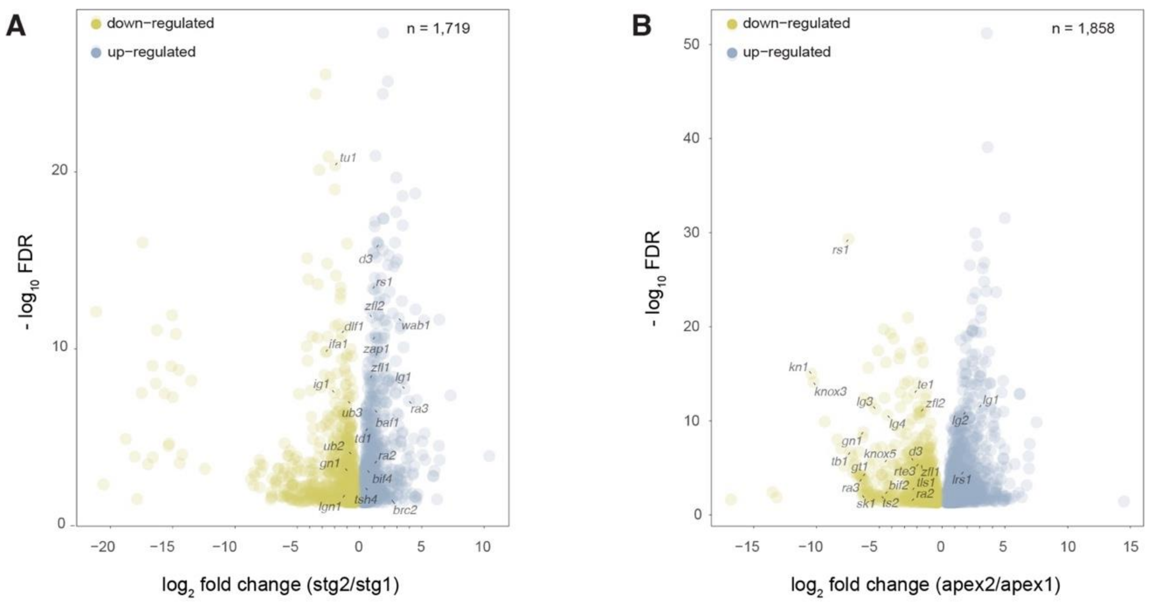

Supplementary Fig. 3. Dynamically expressed genes during tassel branching and ligule differentiation.

A. Volcano plot showing variation in gene expression associated with tassel branch development based on comparing between tassel stages 1 and 2 in B73 normal plants. Genes expressed higher in stage 2 are in blue and those in yellow are decreasing in expression. **B**. Volcano plot representing variation in gene expression associated with ligule differentiation based on the comparison between the shoot apex 1 and 2 samples in B73 normal plants. Genes expressed higher in shoot apex 2 are in blue and those in yellow are decreasing in expression. Only genes with FDR < 0.05 are plotted along the x- ($\log_2$ fold change) and y- ($-\log_{10}$ FDR) axis. Some classical maize genes are annotated.

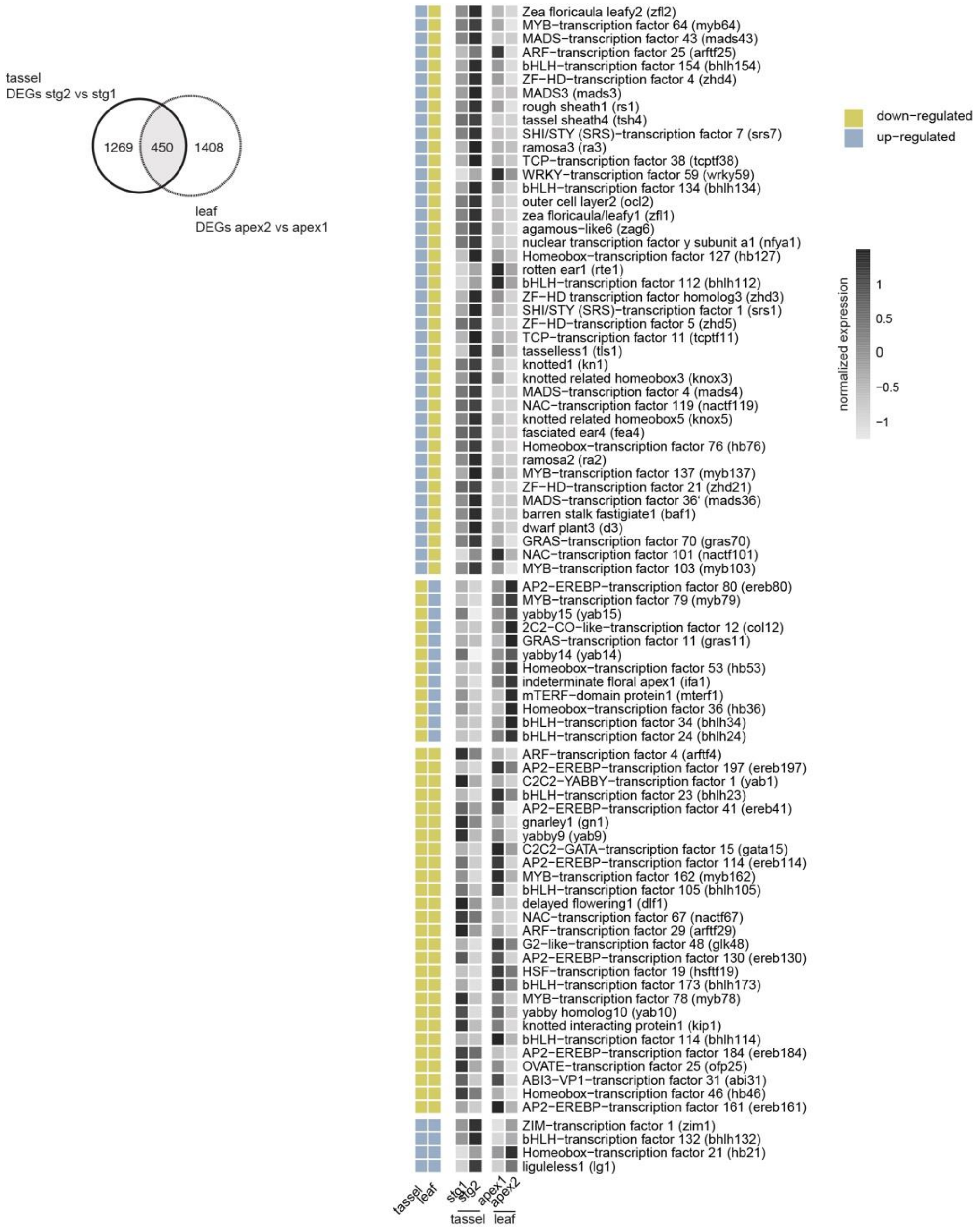

Supplementary Fig. 4. Many genes are differentially expressed in two developmental contexts during tassel branch outgrowth and ligule differentiation.

The heatmap displays relative gene expression profiles of annotated TFs and classical maize genes across both stages of tassel primordia and shoot apices sampled (*n*=86). Genes were differentially expressed in the comparisons between tassel stages 1 and 2 and between shoot apex 1 and 2 with FDR < 0.05 (gray area of the Venn diagram). For each developmental context, DE genes that were up-regulated across developmental time are annotated with a blue box and down-regulated genes annotated with a yellow box.

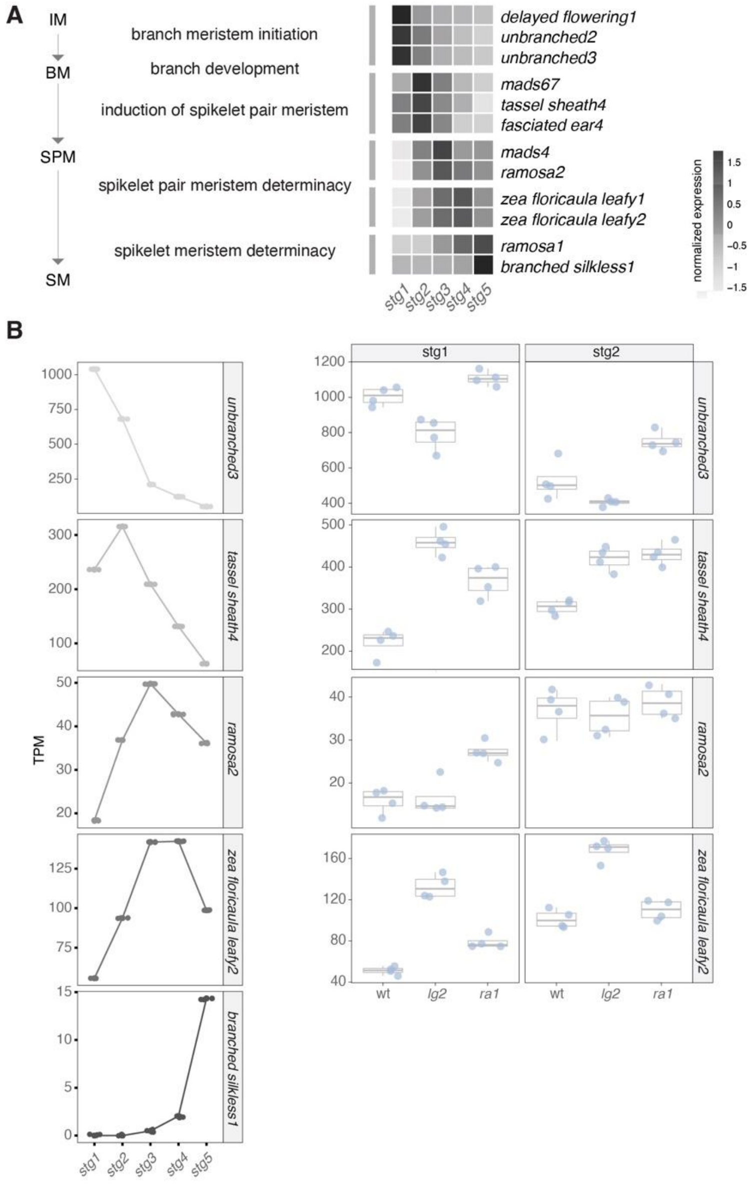

Supplementary Fig. 5. Maize developmental marker genes correspond to meristem identity and determinacy shifts across the tassel developmental gradient.

Expression trajectories of known maize developmental marker genes for meristem identity and determinacy are visualized to assess robustness of the five stage B73 normal tassel developmental gradient. **A**. Heatmap shows gene expression of selected maize inflorescence markers across the five tassel primordia stages in B73. **B**. Peak expression of classical maize genes associated with each stage of meristem development in B73 is shown on the left, and expression profiles for these marker genes in *liguleless2* and *ramosa1* mutant backgrounds are shown on the right. The *branched silkless1* profile is not shown in the mutants since it comes on after stage 2.

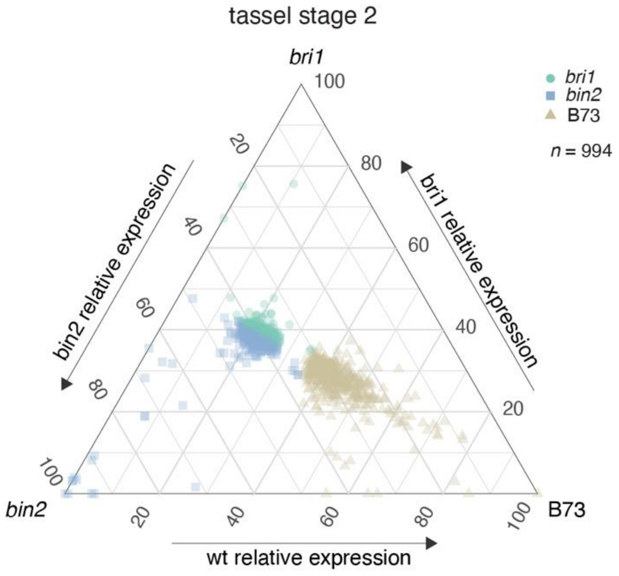

Supplementary Fig. 6. Differences in gene expression of BR mutants compared to B73 controls in stage 2 tassels.

The ternary plot shows the relative expression of genes mis-expressed in common between the two BR mutants compared to B73 at stage 2. Each dot represents a gene and its coordinates indicate relative expression in the three genotypes.

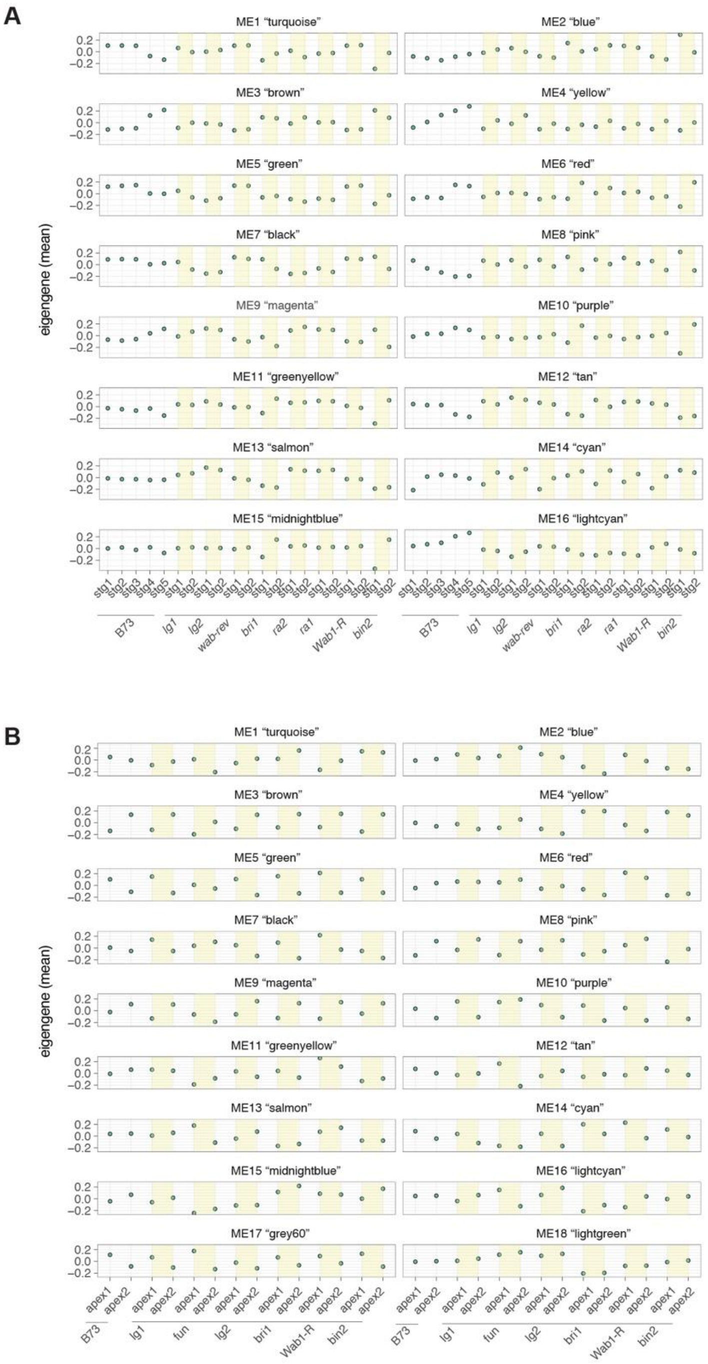

Supplementary Fig 7. Eigengene trajectories of co-expression modules from ‘tassel’ and ‘leaf’ networks.

The expression profile that best fits an average of all genes within a co-expression module is depicted as a Module Eigengene (ME). For each co-expression module in the **A**. ‘tassel’ and **B**. ‘leaf’ GCNs, MEs are depicted by dots in each sample. Each module showed a distinct expression profile, with certain modules characterized by peak expression at specific developmental stages and/or in certain mutant backgrounds.

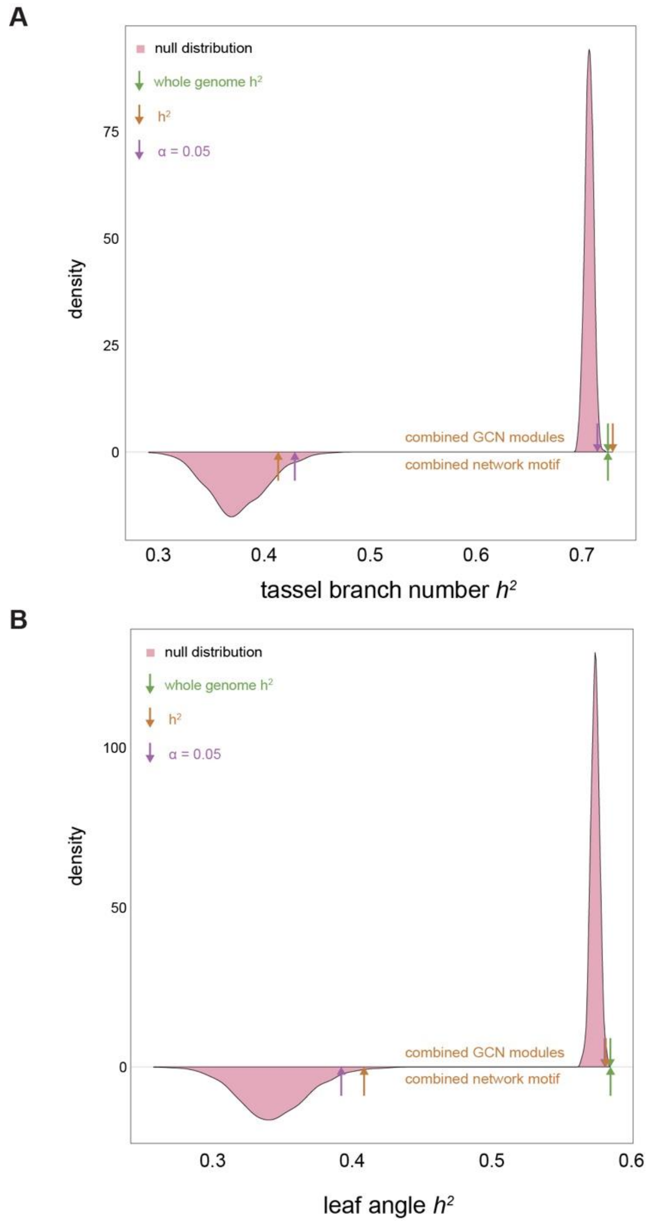

Supplementary Fig. 8. Density plots representing empirical distributions in comparison to whole genome h^2 and TBN and LA h^2.
TBN h^2. Above 0 density represents the null distribution (purple arrow shows the 95th percentile) in comparison to h^2 explained by the selected co-expressed network modules (orange arrow) and whole genome h^2 (green arrow). Below 0 density represents null distribution (purple arrow shows the 95th percentile) in comparison to h^2 explained by the combined network motif (orange arrow) and whole genome h^2 (green arrow). **B**. LA h^2. Upper panel represents the null distribution (purple arrow shows the 95th percentile) in comparison to h^2 explained by the selected co-expressed network modules (orange arrow) and whole genome h^2 (green arrow). Lower panel represents null distribution (purple arrow shows the 95th percentile) in comparison to h^2 explained by the combined network motif (orange arrow) and whole genome h^2 (green arrow). Source data are provided as a Source Data file.

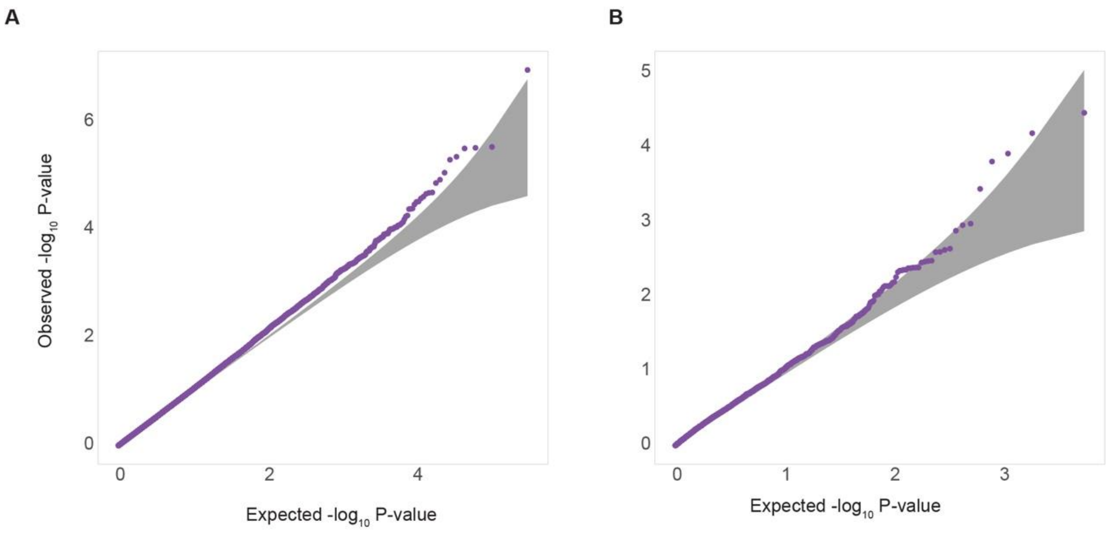

Supplementary Fig. 9. Quantile-Quantile plots from the multivariate GWAS for TBN and LA.

Distribution of observed versus expected –$\log_{10}$ P-values from the multivariate association using **A**. combined gene co-expression modules and **B**. combined network motifs.

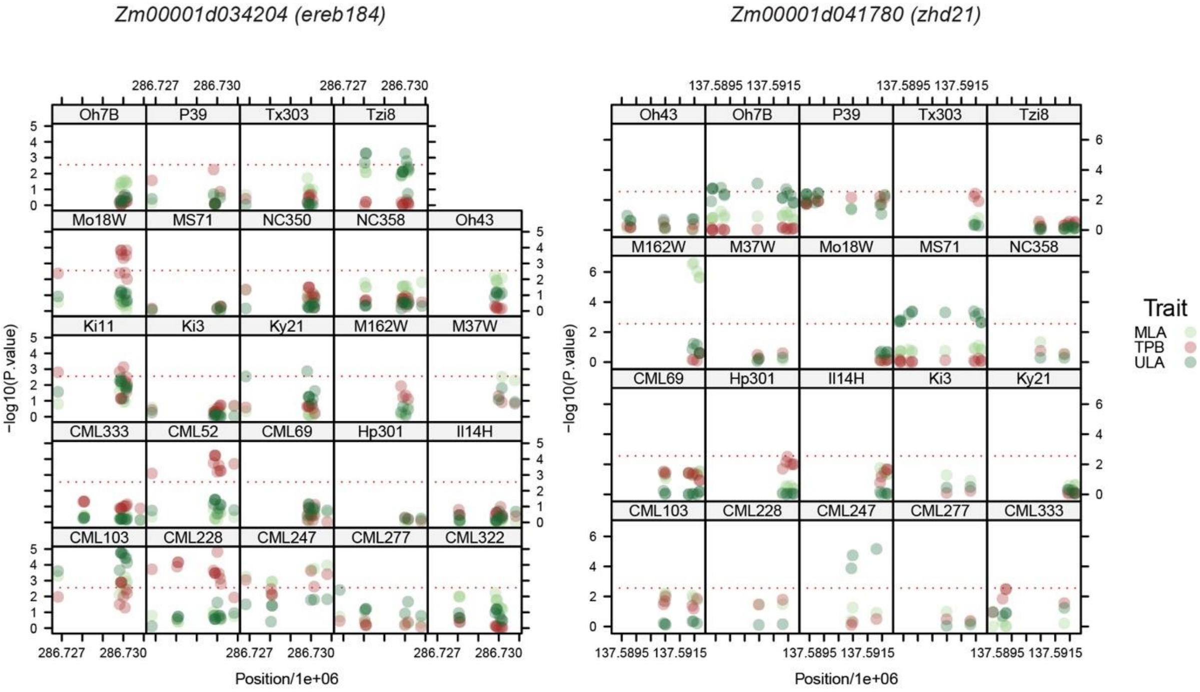

Supplementary Fig. 10. Candidate gene associations in the Maize Nested Association Mapping (NAM) biparental families.

Association results from a generalized linear model using SNPs assigned to *ereb184* (left) and *zhd21* (right) segregating in NAM biparental families for TBN and LA. Association tests were conducted independently in each family. Each SNP, color-coded by phenotype, is plotted by its –$\log_{10}$ P-value (y-axis) and mb position (x-axis). All marker coordinates correspond to the B73 maize reference genome v4. Red lines represent a Bonferroni threshold based on the total number of segregating SNPs for a given gene: 20 for *ereb184* and 22 for *zhd21*.

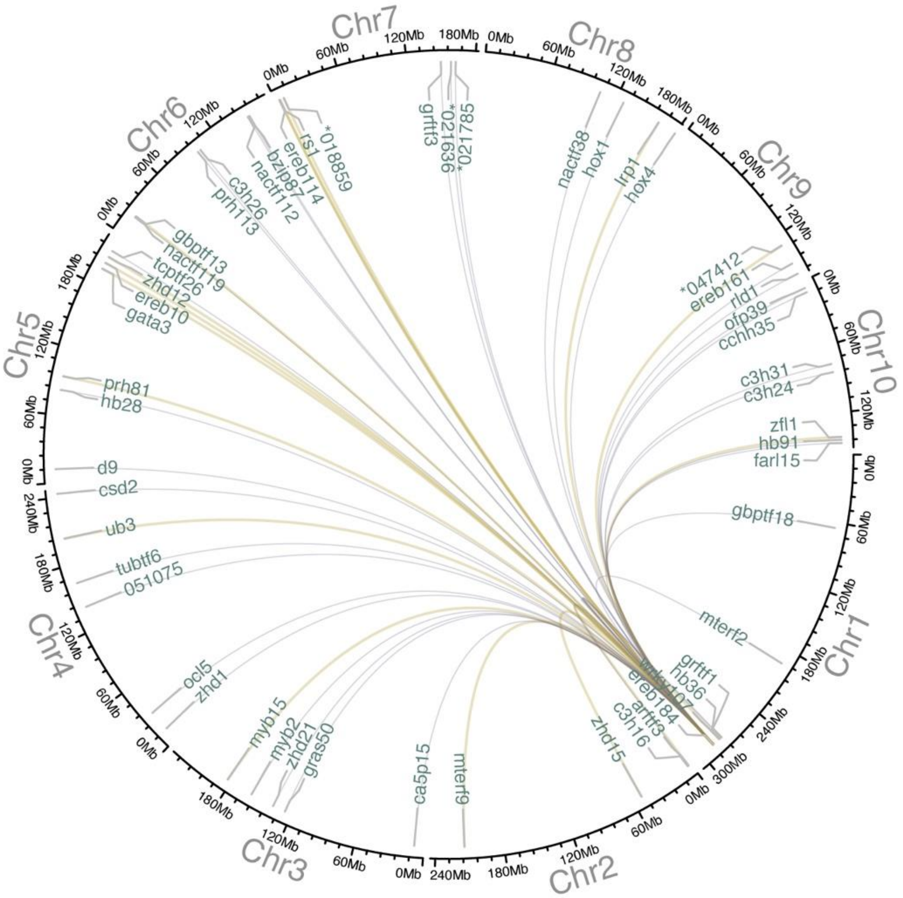

Supplementary Fig. 11. Depiction of statistical genetic interactions predicted for ereb184 with the top recurrent TFs within three-node network motifs.

Based on makers within the genomic windows defined as ± 2 kb from the transcriptional start site (TSS) and transcriptional termination site (TTS) of *ereb184* and the recurrent TFs from 3-node motifs, statistical genetic interactions were predicted. These interactions are represented as links between genes located on a circular visualization of the maize genome. Grey and tan links represent significant interactions for the traits TBN, LA and PhPC2. Tan links highlight statistical interactions that are supported by the GRN data.

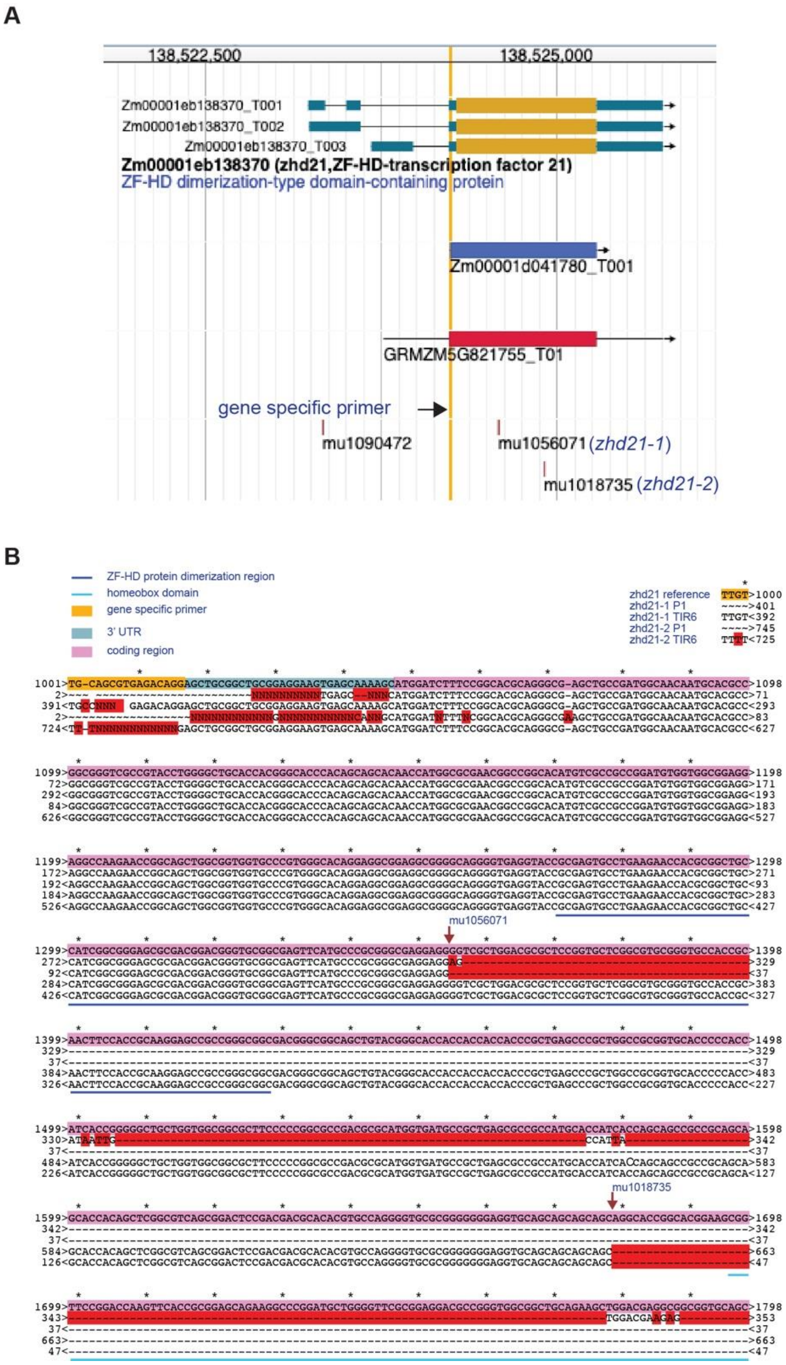

Supplementary Fig. 12. Graphical representation of Mutator (Mu) transposon insertions at the *zhd21* locus.

A. Genome browser representation of the *zhd21* locus in the maize reference genome NAM v5. The *zhd21-1* (*mu1056071*) and *zhd21-2* (*mu1018735*) alleles are indicated as red vertical bars relative to the gene coordinates. The gene-specific primer used in the genotyping analysis is represented as a vertical yellow bar. **B**. Sequencing alignment of the PCR products using the gene-specific primer in combination with the Mu-TIR primer for *zhd21-1* and *zhd21-2*.

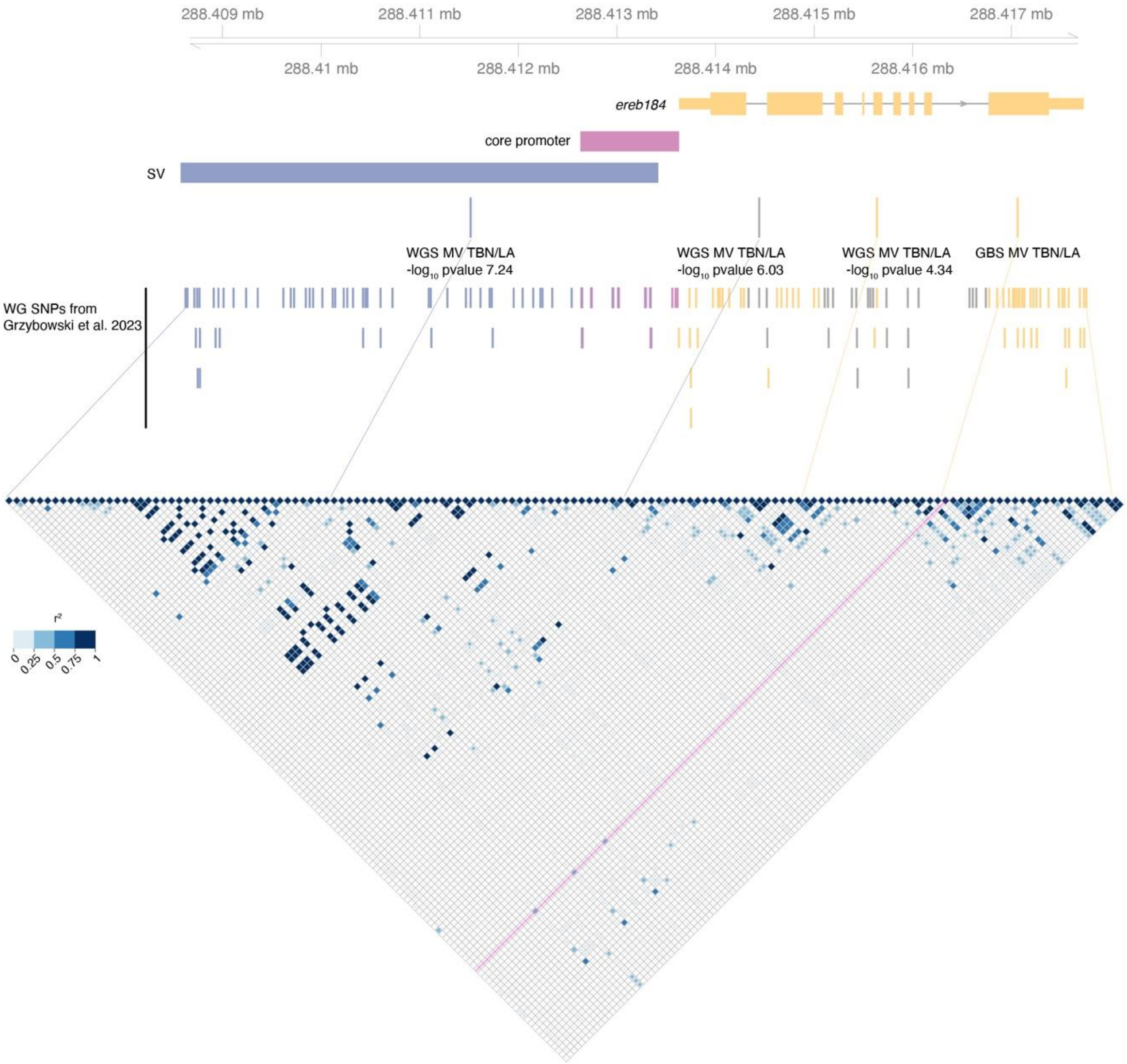

Supplementary Fig. 13. Local linkage disequilibrium (LD) heatmap at the *ereb184* locus. Genome browser representation of the *ereb184* locus in the B73 maize reference genome v4. The upper panel represents the gene structure and the upstream regulatory region, including the predicted core promoter and the structural variation (SV) of approximately 5 kb. Results of the multivariate candidate gene association analysis for TBN/LA using whole-genome (WG) SNPs are reported as bars with their $-\log_{10}$ P-values next to the GBS network-guided GWAS marker. All WG SNPs located in the genomic region are represented as bars and are color-coded based on the genomic region in which they reside. The local LD plot shown in the bottom panel represents pairwise LD measurements between WG SNPs within the genomic region. The purple line highlights the LD between the GBS SNPs and the SV located upstream. Source data are provided as a Source Data file.

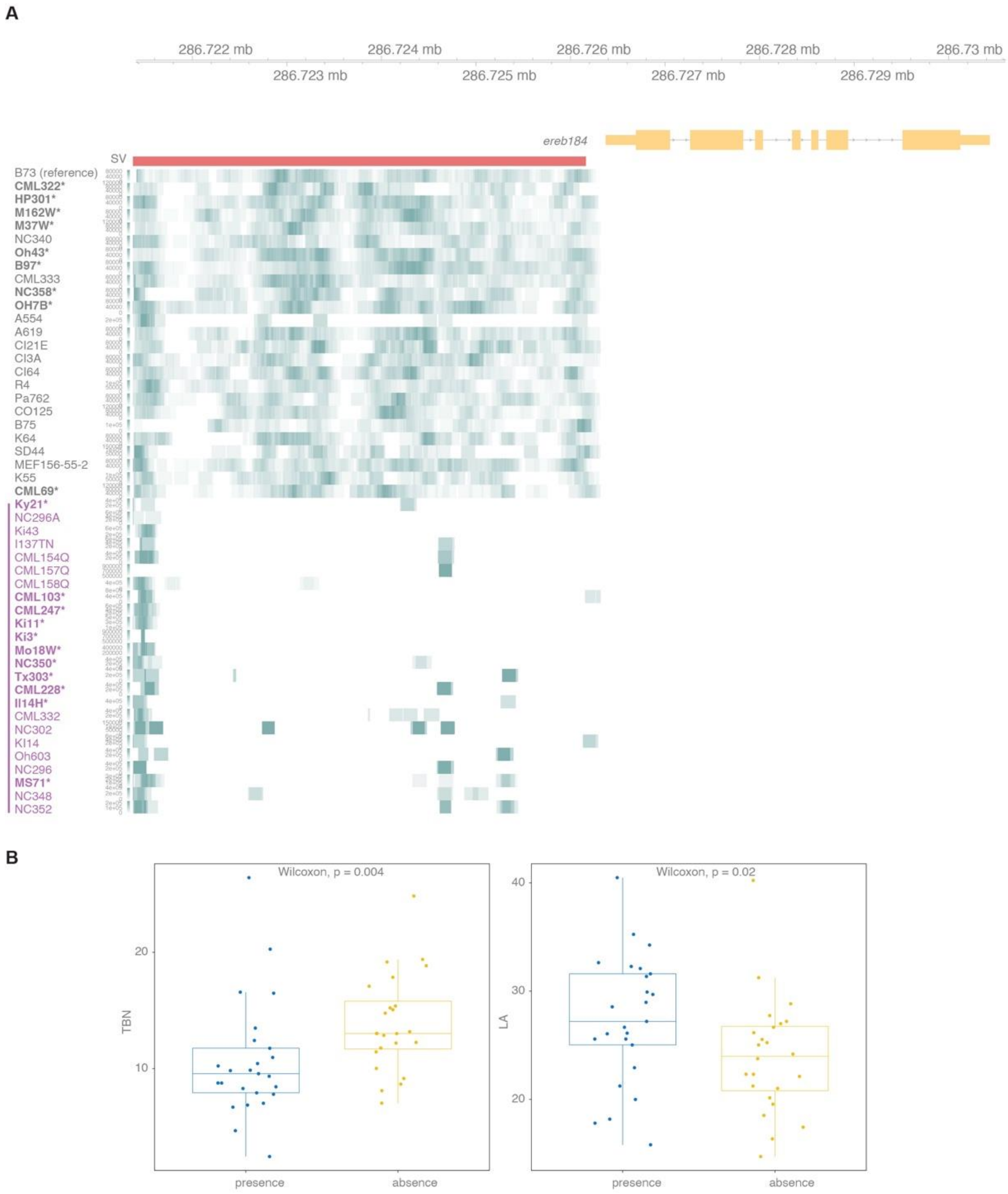

Supplementary Fig. 14. Graphical representation of the presence/absence variation (PAV) of the SV at the *ereb184* locus.

Graphical representation of the SV at the *ereb184* locus based on existing sequencing data [13]. **A**. Genomic view (maize reference AGPv4) of forty-nine inbred lines chosen based on genetic similarity (kinship matrix) relative to NAM founders shows data supporting PAV of the SV at the ereb184 locus are reported as heat-map tracks representing the sequencing coverage (normalized based on Bins Per Million mapped (BPM) reads with bin size= 1). NAM lines are highlighted in bold and marked with a star. **B**. The effect of structural variation (SV) presence/absence in the promoter region of *ereb184* across 49 accessions (upper panel) on leaf angle and tassel branch number traits. Source data are provided as a Source Data file.

Supplementary references

1. Becraft, P. W., Bongard-Pierce, D. K., Sylvester, A. W., Poethig, R. S. & Freeling, M. The *liguleless-1* gene acts tissue specifically in maize leaf development. *Dev. Biol.* **141**, 220–232 (1990).
2. Moreno, M. A., Harper, L. C., Krueger, R. W., Dellaporta, S. L. & Freeling, M. *liguleless1* encodes a nuclear-localized protein required for induction of ligules and auricles during maize leaf organogenesis. *Genes Dev.* **11**, 616–628 (1997).
3. Walsh, J., Waters, C. A. & Freeling, M. The maize gene *liguleless2* encodes a basic leucine zipper protein involved in the establishment of the leaf blade-sheath boundary. *Genes Dev.* **12**, 208–218 (1998).
4. Harper, L. & Freeling, M. Interactions of *liguleless1* and *liguleless2* function during ligule induction in maize. *Genetics* **144**, 1871–1882 (1996).
5. Kellogg, E. A. Floral displays: genetic control of grass inflorescences. *Curr. Opin. Plant Biol.* **10**, 26–31 (2007).
6. Vollbrecht, E., Springer, P. S., Goh, L., Buckler, E. S., 4th & Martienssen, R. Architecture of loral branch systems in maize and related grasses. *Nature* **436**, 1119–1126 (2005).
7. Eveland, A. L. *et al.* Regulatory modules controlling maize inflorescence architecture. *Genome Res.* **24**, 431–443 (2014).
8. Lewis, M. W. *et al.* Gene regulatory interactions at lateral organ boundaries in maize. *Development* **141**, 4590–4597 (2014).
9. Bai, F., Reinheimer, R., Durantini, D., Kellogg, E. A. & Schmidt, R. J. TCP transcription factor, BRANCH ANGLE DEFECTIVE 1 (BAD1), is required for normal tassel branch angle formation in maize. *Proc. Natl. Acad. Sci. U. S. A.* **109**, 12225–12230 (2012).
10. Bortiri, E. *et al.* ramosa2 encodes a LATERAL ORGAN BOUNDARY domain protein that determines the fate of stem cells in branch meristems of maize. *Plant Cell* **18**, 574–585 (2006).
11. Vajk, A. H. The role of FUN in sexual differentiation and leaf development in *Zea mays*. (UC Berkeley, 2019). https://escholarship.org/uc/item/29r2x7hh
12. Kir, G. Regulation of shoot development in maize via brassinosteroid signaling. (Iowa State University, 2015). https://dr.lib.iastate.edu/entities/publication/a1340a36-5af0-4c4c-9a67-b09d5083c99f

13. Song, B. et al. Conserved noncoding sequences provide insights into regulatory sequence and loss of gene expression in maize. *Genome Res.* **31**, 1245–1257 (2021).
