# Supplementary material for: Regulatory variation controlling architectural pleiotropy in maize
Source: Nat Commun. 2025 Mar 3;16:2140. doi: 10.1038/s41467-025-56884-w (PMC11876617; doi:10.1038/s41467-025-56884-w)
Supplement: Supplementary file 3 — Description of Additional Supplementary Files [file 41467_2025_56884_MOESM3_ESM.pdf]

Description of Additional Supplementary Files

Supplementary Data 1: Gene expression in TPM across 140 RNA-seq samples.
Supplementary Data 2: DE genes with FDR < 5% derived from the comparison of B73 stage 2 tassel primordia vs. stage 1.
Supplementary Data 3: DE genes with FDR < 5% derived from the comparison of B73 shoot apex 2 vs. shoot apex 1.
Supplementary Data 4: GO terms associated with DE genes derived from the comparison of B73 stage 2 tassel primordia vs. stage 1.
Supplementary Data 5: GO terms associated with the DE genes derived from the comparison of B73 shoot apex 2 vs. shoot apex 1.
Supplementary Data 6: Genes that are DE between tassel stage 1 and stage 2 and shared between the BR mutants compared to B73 control.
Supplementary Data 7: Relative expression of genes commonly DE in the two BR mutants along the tassel stage 1 to stage 2 developmental gradient compared to the B73 control.
Supplementary Data 8: GO and TF enrichment analysis of genes commonly DE in the two BR mutants between tassel stage 1 and stage 2 development compared to the B73 control.
Supplementary Data 9: Summary of gene co-expression modules from the 'tassel' and 'leaf' context-specific networks.
Supplementary Data 10: Maize genes grouped based on the 'tassel' and 'leaf' gene co-expression modules.
Supplementary Data 11: Gene co-expression module preservation matrix between 'tassel' and 'leaf' networks.
Supplementary Data 12: GO terms overrepresented within 'tassel' and 'leaf' GCN modules.
Supplementary Data 13: GO terms overrepresented within 'tassel' and 'leaf' GCN preserved sub-modules.
Supplementary Data 14: TF enrichment analysis of the predicted regulators of *liguleless 2* in the leaf GRN shown in Figure 2c.
Supplementary Data 15: i) Summary statistics of the three-node network motifs annotated in the GRNs. ii) Top one-hundred recurrent TFs within the three-node network motifs.
Supplementary Data 16: GEBVs and BLUPs of the elected Ames diversity lines.
Supplementary Data 17: Pearson correlations of phenotypic traits.
Supplementary Data 18: Module-trait correlation of the sixteen modules identified in the comprehensive GCN.
Supplementary Data 19: Gene co-expression module heritabilities (h^2) for leaf angle (LA) and tassel branch number (TBN).
Supplementary Data 20: GWAS SNP-trait associations for LA, TBN, PhPC1 and PhPC2 from the single-trait approach using two sets of markers.
Supplementary Data 21: GO terms associated with genes proximal to significant SNP-trait associations from the single-trait GWAS for PhPC2.
Supplementary Data 22: Multi-trait GWAS SNP-trait associations for LA, TBN using two sets of markers.
Supplementary Data 23: Predicted direct gene targets of EREB184 and ZHD21 in the 'leaf' GRN.

Supplementary Data 24: Statistical genetic interaction predictions between *ereb184 and* top recurrent TFs within three-node network motifs.
Supplementary Data 25: Single-trait GWAS results for LA based markers subset around sorghum orthologs of the highly connected three-node motifs in the maize networks.
Supplementary Data 26: GO terms associated with genes in position Z of *ereb184*-controlled FFLs in the 'leaf' network.
