# Supplementary material for: Regulatory variation controlling architectural pleiotropy in maize
Source: Nat Commun. 2025 Mar 3;16:2140. doi: 10.1038/s41467-025-56884-w (PMC11876617; doi:10.1038/s41467-025-56884-w)
Supplement: Supplementary file 30 — Reporting Summary [file 41467_2025_56884_MOESM30_ESM.pdf]

nature portfolio

Corresponding author(s): Andrea L. Eveland

Last updated by author(s): Jan 25, 2025

Reporting Summary

Nature Portfolio wishes to improve the reproducibility of the work that we publish. This form provides structure for consistency and transparency in reporting. For further information on Nature Portfolio policies, see our Editorial Policies and the Editorial Policy Checklist.

Statistics

For all statistical analyses, confirm that the following items are present in the figure legend, table legend, main text, or Methods section.

n/a	Confirmed	
☐	☒	The exact sample size (*n*) for each experimental group/condition, given as a discrete number and unit of measurement
☐	☒	A statement on whether measurements were taken from distinct samples or whether the same sample was measured repeatedly
☐	☒	The statistical test(s) used AND whether they are one- or two-sided<br>*Only common tests should be described solely by name; describe more complex techniques in the Methods section.*
☐	☒	A description of all covariates tested
☐	☒	A description of any assumptions or corrections, such as tests of normality and adjustment for multiple comparisons
☐	☒	A full description of the statistical parameters including central tendency (e.g. means) or other basic estimates (e.g. regression coefficient) AND variation (e.g. standard deviation) or associated estimates of uncertainty (e.g. confidence intervals)
☐	☒	For null hypothesis testing, the test statistic (e.g. *F*, *t*, *r*) with confidence intervals, effect sizes, degrees of freedom and *P* value noted<br>*Give P values as exact values whenever suitable.*
☒	☐	For Bayesian analysis, information on the choice of priors and Markov chain Monte Carlo settings
☒	☐	For hierarchical and complex designs, identification of the appropriate level for tests and full reporting of outcomes
☐	☒	Estimates of effect sizes (e.g. Cohen's *d*, Pearson's *r*), indicating how they were calculated

Our web collection on statistics for biologists contains articles on many of the points above.

Software and code

Policy information about availability of computer code

Data collection	Published phenotype data used for genomic prediction were downloaded from panzea.org<br>Maize TFs annotation was downloaded from grassius.org<br>Maize GO annotation was downloaded from GOMAP doi.org/10.7946/P2M925<br>GO annotation was downloaded from QuickGO https://www.ebi.ac.uk/QuickGO<br>Sorghum-maize syntenic orthologs were downloaded from https://figshare.com/articles/dataset/Grass_Syntenic_Gene_List_sorghum_v3_maize_v3_4_with_teff_and_oropetium_v2/7926674/1<br>Sorghum reference files were downloaded from Phytozome v12.1<br>Maize reference files were downloaded from Ensembl Plants release 34
Data analysis	RNA-seq, ATAC-seq:<br>Stand alone programs: Samtools v1.9, Cutadapt v2.3, TrimGalore v0.6.2, Salmon v1.4.0, bowtie2 v2.4.5, MACS2 v2.1.2<br>R packages: tximport, DESeq2, WGCNA, igraph, GeneOverlap, GENIE3, GenomicRanges<br>Association analyses:<br>Stand alone programs: ASReml-R 4.1, CrossMap v0.3.7, VCFtools v0.1.12, REML, LDAK v5.1, GAPIT v3, GEMMA v0.98.6, PLINK v1.9.

For manuscripts utilizing custom algorithms or software that are central to the research but not yet described in published literature, software must be made available to editors and reviewers. We strongly encourage code deposition in a community repository (e.g. GitHub). See the Nature Portfolio guidelines for submitting code & software for further information.

Data

Policy information about availability of data

All manuscripts must include a data availability statement. This statement should provide the following information, where applicable:

- Accession codes, unique identifiers, or web links for publicly available datasets
- A description of any restrictions on data availability
- For clinical datasets or third party data, please ensure that the statement adheres to our policy

Raw and processed data are available through NCBI Gene Expression Omnibus (GEO) database using the identification number GSE180593. Genotype data are available at panzea.org. Scripts used in this study are available and archived online for download on figshare: doi:10.6084/m9.figshare.27984821

Research involving human participants, their data, or biological material

Policy information about studies with human participants or human data. See also policy information about sex, gender (identity/presentation), and sexual orientation and race, ethnicity and racism.

Reporting on sex and gender	Our study did not involve human participants, their data, or biological material
Reporting on race, ethnicity, or other socially relevant groupings	Our study did not involve human participants, their data, or biological material
Population characteristics	Our study did not involve human participants, their data, or biological material
Recruitment	Our study did not involve human participants, their data, or biological material
Ethics oversight	Our study did not involve human participants, their data, or biological material

Note that full information on the approval of the study protocol must also be provided in the manuscript.

Field-specific reporting

Please select the one below that is the best fit for your research. If you are not sure, read the appropriate sections before making your selection.

☒ Life sciences ☐ Behavioural & social sciences ☐ Ecological, evolutionary & environmental sciences

For a reference copy of the document with all sections, see nature.com/documents/nr-reporting-summary-flat.pdf

Life sciences study design

All studies must disclose on these points even when the disclosure is negative.

Sample size	Phenotyping: The number of lines chosen per year (n=425) allowed us to phenotype two replicate blocks within our field dimensions, providing good power for association analyses.<br>RNA-seq: Based on our experience, four biological replicates is sufficient to capture the biological variation within sample types
Data exclusions	RNA-seq: only one sample derived from the lg1-R stage 1 tassel (replicate 2) was excluded from the network analyses because sequencing coverage was below the threshold.<br>Phenotyping: a small number of values were not included because of recording errors.
Replication	Phenotyping: The field experimental design was a Randomized Complete Block Design with two blocks each year.<br>RNA-seq: Four biological replicates represented each sample. All attempts at replication were successful
Randomization	Phenotyping: Genotypes were randomly assigned within blocks.<br>RNA-seq: Samples were multiplexed and randomized before sequencing within Illumina lanes.
Blinding	In our study there were no treatment groups and therefore blinding was not relevant

Reporting for specific materials, systems and methods

We require information from authors about some types of materials, experimental systems and methods used in many studies. Here, indicate whether each material, system or method listed is relevant to your study. If you are not sure if a list item applies to your research, read the appropriate section before selecting a response.

Materials & experimental systems

n/a	Involved in the study
☒	☐ Antibodies
☒	☐ Eukaryotic cell lines
☒	☐ Palaeontology and archaeology
☒	☐ Animals and other organisms
☒	☐ Clinical data
☒	☐ Dual use research of concern
☐	☒ Plants

Methods

n/a	Involved in the study
☒	☐ ChIP-seq
☒	☐ Flow cytometry
☒	☐ MRI-based neuroimaging

Plants

Seed stocks

UniforMu lines (ID: mu1022277, ID: mu1056071, ID mu1018735) were ordered from the Maize Genetics COOP stock center https://www.maizegdb.org/stock_catalog
Maize inbred lines (n=1064) were ordered from USDA-ARS Germplasm Resources Information Network (GRIN) https://www.ars-grin.gov/ The accession IDs can be found in Supplementary table 16

Novel plant genotypes

There were no novel plant genotypes used in this study

Authentication

Zhd alleles were backcrossed into the W22 inbred line for at least two generations. PCR was used to confirm the presence of the transposon insertions in each line.
